# Double and Compound Fracture of the Thigh

**Published:** 1856-03

**Authors:** David Prince

**Affiliations:** Jacksonville, Ill.


ARTICLE IV.—*Double and Compound Fracture of the Thigh. Fatal after* 74 *days.* By DAVID PRINCE, M.D., Jacksonville, Ill.

Wm. Wilkinson, æt. 54, of good constitution, having had little or no sickness during his life, was (Nov. 20, 1855,) digging around a post under a shed, for the purpose of replacing it by a new one, when the upper part of the structure came down upon him, throwing him down, with his right thigh bent over a log, while the foot of the same side became fixed by some of the material falling upon it. His right elbow was dislocated, his right thigh broken obliquely in the middle third, and also in the lower third obliquely, the lower point of the middle fragment projecting through the skin in the line of the external hamstring.

Visited him four hours after the injury; his temperature was diminished and his pulse quickened by the shock, but after the adjustment and application of apparatus, he soon reacted. Rose's double inclined splint was first applied, not on account of a preference for the bent position, but for convenience. After two days a straight splint was applied, composed of a thin board, three inches wide, extending from the trochanter major to three inches below the foot, and forked at either extremity. A belt four inches wide around the upper part of the thigh, with straps and buckles, and a loop to receive one of the forks of the splint, constituted the counter extending support, while the extending shoe and extending bar of Járvis' adjuster, constituted the extending apparatus.

The limb, bound with the roller below the knee, and the many tailed bandage above the knee, in the usual way, was laid upon the splint accompanying Jarvis' adjuster, well cushioned, and a narrow splint corresponding with the one outside, but shorter, was placed upon the inside of the limb, cushioned, and the whole secured by straps applied around all the three splints.

One of the objects of this communication is to notice some difficulties in the application of Dr. Jarvis' Adjuster to fractures. It is not an unnatural ambition in one who invents an instrument really useful for certain purposes, to seek to endow it with universal applicability, and this very justifiable ambition seems to have taken possession of Dr. Jarvis. His apparatus is certainly a very valuable one for dislocations, but for fractures requiring long continued extension and counter extension, the novice who attempts to follow literally the directions of Dr. Jarvis, will probably find himself soon foiled by ulcerations in the groin and upon the instep, or above the condyles of the femur, or wherever else the pressure comes. The pressure by the rollers for the groin and pads for the condyles of the femur press upon too small an extent of surface to be borne day after day, as in fractures, where it is necessary to keep up constant tension. The thigh fork of this apparatus is also inapplicable in fractures, with the patient lying on his back, the lower extremity of it being a source of permanent uneasiness, and being in the way of the broad splint upon which the limb is intended to rest. The broad belt above mentioned obviated these difficulties, allowing the extending splint to be placed upon the outer side of the limb. The extending bar of this apparatus attached to the lower end of the same splint, allowed of regular, easy, and effectual extension. The only difficulty here, was to get a good point of application for the extension. This was found in the application of a belt of adhesive strips to the middle portion of the leg, with loops into which bands were thrust, extending thence over the cross piece of the extending bar. By this arrangement there was no difficulty in keeping the injured leg of the same length with the sound one, and without discomfort to the patient.

During the first few days, the patient's diet was diminished in richness and quantity from that of health, but afterwards generous diet, with wine and quinine, were enjoined. During the second week of confinement, some symptoms of intermittent fever manifested themselves; but during the third and fourth weeks the appetite was good and the spirits cheerful, with good pulse. During the fifth week, however, the appetite diminished, and the pulse became more frequent; the extremities inclining to be cold, and neuralgic or rheumatic pains in various parts of the body. In the sixth week, there came a discharge of pus through the perforation originally made by the extremity of fractured bone, and from this time till death, from eight to sixteen ounces of pus were discharged daily through this opening. Towards the last, sloughing occurred, exposing considerable portions of the neighboring muscles—his appetite entirely failed, and he finally died, gradually exhausted and emaciated to the last degree.

No diarrhœa at any time complicated the case, and no discharge subsequent to the original bleeding appeared from the lacerated skin, until the flow of pus in the sixth week. No bony consolidation took place in either fracture.

To what was the failure owing in this case? Evidently to the want of recuperative power in the constitution of the patient, for though so nice had been the balance that no disease had ever spontaneously occurred, yet when once the balance was disturbed there was no power to restore it.

